# A role of TRIM59 in pulmonary hypertension: modulating the protein ubiquitylation modification

**DOI:** 10.1186/s12967-023-04712-4

**Published:** 2023-11-17

**Authors:** Yingli Liu, Li Zhu, Yue Ming, Zhuhua Wu, Lili Zhang, Qi Chen, Yong Qi

**Affiliations:** 1 Department of Pulmonary and Critical Care Medicine, Zhengzhou University People’s Hospital, Henan Provincial People’s Hospital, Zhengzhou, People’s Republic of China; 2 Academy of Medical Science, Zhengzhou University, Zhengzhou, People’s Republic of China; 3 Department of Pulmonary and Critical Care Medicine, Henan University People’s Hospital, Henan Provincial People’s Hospital, Zhengzhou, People’s Republic of China; 4 Department of Pulmonary and Critical Care Medicine, Zhengzhou University People’s Hospital, Henan Provincial People’s Hospital, Henan University People’s Hospital, Zhengzhou, People’s Republic of China

**Keywords:** TRIM59, Pulmonary hypertension, Ubiquitylation modification, YAP1/TEAD4, Pulmonary artery smooth muscle cells

## Abstract

**Background:**

Pulmonary hypertension (PH), an infrequent disease, is characterized by excessive pulmonary vascular remodeling and proliferation of pulmonary artery smooth muscle cells (PASMCs). However, its underlying molecular mechanisms remain unclear. Uncovering its molecular mechanisms will be beneficial to the treatment of PH.

**Methods:**

Differently expressed genes (DEGs) in the lung tissues of PH patients were analyzed with a GEO dataset GSE113439. From these DEGs, we focused on TRIM59 which was highly expressed in PH patients. Subsequently, the expression of TRIM59 in the pulmonary arteries of PH patients, lung tissues of PH rat model and PASMCs cultured in a hypoxic condition was verified by quantitative real-time PCR (qPCR), western blot and immunohistochemistry. Furthermore, the role of TRIM59 in PAMSC proliferation and pathological changes in PH rats was assessed via gain-of-function and loss-of-function experiments. In addition, the transcriptional regulation of YAP1/TEAD4 on TRIM59 was confirmed by qPCR, western blot, luciferase reporter assay, ChIP and DNA pull-down. In order to uncover the underlying mechanisms of TRIM59, a protein ubiquitomics and a CoIP- HPLC–MS/MS were companied to identify the direct targets of TRIM59.

**Results:**

TRIM59 was highly expressed in the pulmonary arteries of PH patients and lung tissues of PH rats. Over-expression of TRIM59 accelerated the proliferation of PASMCs, while TRIM59 silencing resulted in the opposite results. Moreover, TRIM59 silencing mitigated the injuries in heart and lung and attenuated pulmonary vascular remodeling during PH. In addition, its transcription was positively regulated by YAP1/TEAD4. Then we further explored the underlying mechanisms of TRIM59 and found that TRIM59 overexpression resulted in an altered ubiquitylation of proteins. Accompanied with the results of CoIP- HPLC–MS/MS, 34 proteins were identified as the direct targets of TRIM59.

**Conclusion:**

TRIM59 was highly expressed in PH patients and promoted the proliferation of PASMCs and pulmonary vascular remodeling, thus contributing to the pathogenesis of PH. It is indicated that TRIM59 may become a potential target for PH treatment.

## Introduction

Pulmonary hypertension (PH) is an infrequent disease characterized by small pulmonary artery obliteration, leading to pulmonary arterial pressure elevation and right heart failure. Recent treatment for PH predominantly targets vasoactive mediators, including endothelin1 and prostacyclin, which transduce signals through G-protein-coupled receptors [1]. Whereas, the employment of these treatments is limited by their action as pulmonary vasodilators, but no effects on pulmonary vascular remodeling which is the cornerstones of disease progression [2]. Thus, exploring the underlying molecular mechanisms of pulmonary vascular remodeling during PH is significant to the development of novel PH treatment.

The pathological characteristics of PH includes dysfunction of pulmonary arterial endothelial cells, proliferation of pulmonary artery smooth muscle cells (PASMCs), vasoconstriction and thrombosis in situ [3]. Among these characteristics, excessive PASMC proliferation leads to the neointima formation, resulting in progressive pulmonary artery obliteration, arisen blood resistance and ultimately right heart failure. Literature review showed that targeting genes which contribute to the excessive proliferation of PASMCs can relieve PH progress [4–6]. However, the exact molecular mechanisms of excessive PASMC proliferation remain unclear. Revealing the underly molecular mechanism of excessive PASMC proliferation may be of benefit to the treatment of PH.

Tripartite motif containing 59 (TRIM59), an E3 ubiquitin ligase which links ubiquitin to targeted proteins, participates in the regulation of cell growth and inflammation [7–9]. It mediates the ubiquitination of downstream proteins, such as Beclin 1, protein phosphatase 1B and glutathione peroxidase 4, thus performing its function in biological processes [8, 10, 11]. We previously analyzed the gene expression profiling of lung tissues from PH patients in GEO database (https://www.ncbi.nlm.nih.gov/geo/) and found that TRIM59 was highly expressed in PH patients. However, its role in PH as well as the underlying mechanism remains unknown.

Yes-associated protein (YAP1) is a core effector downstream the Hippo signaling pathway, which is closely associated with diseases of respiratory system including PH [12]. It was pointed out that YAP1 was highly expressed in the PH model [13, 14] and the inactivation of YAP1 ameliorated PH [12, 15]. YAP1 acts as a transcriptional coactivator to modulate the transcription of downstream genes. Interestingly, TEA domain transcription factor (TEAD) 4, A DNA-anchor protein, which is needed by YAP1 to form a transcription complex in order to modulate the transcription of target genes as well as to perform its role in cell growth [16]. According to the analysis from Jaspar (https://jaspar.genereg.net/), in the promoter region of TRIM59, there were potential binding sites of TEAD4. We wondered whether the transcription of TRIM59 was modulated by YAP1/TEAD4.

In this study, we showed that TRIM59, whose transcription was promoted by YAP1/TEAD4, modulated the protein ubiquitination modification in PASMCs, accelerating the proliferation of PASMCs, thus contributing to the occurrence of PH. This study provided evidence for the potential role of TRIM59 in PH. It is indicated that TRIM59 may become a potential target for the treatment of pulmonary vascular remodeling during PH.

## Materials and methods

### GEO data analysis

GSE113439 contains the gene expression profile of lung tissues from 15 PH patients and 11 normal controls. The gene expression was analyzed with GEO2R, a built-in analysis module.

### Human samples of PH

The pulmonary arteries samples of 8 patients with PH and 4 controls were obtained. The criteria of PH diagnosis in this study were based on the 2022 ESC/ERS Guidelines for Pulmonary Hypertension [17]. PH is defined by a mean pulmonary arterial pressure > 20 mmHg at rest. Written informed consents were obtained from patients. This study was conducted in accordance with the Declaration of Helsinki, and approved by the Medical Ethics Committee of Henan Provincial People's Hospital [Ethical Review 2021(60)].

### Experimental animals

Six-week-old male Sprague Dawley rats were obtained from Liaoning Changsheng Biotechnology Co., Ltd (Benxi, China) and fed in a standard environment (temperature 21–23 °C, humidity 45–55%, 12 h light/12 h dark cycles, free access to food and water). All animal experiments were performed in accordance with the Guidelines for the Care and Use of Laboratory Animals and approved by the Henan Institute for Food and Drug Control (No. YXKC2020005-1).

Experiment 1: Rats were divided into 2 groups: Control (Con, n = 6) and PH model (SuHx, n = 6). Rats in the SuHx group received a single injection of Sugen-5416 (20 mg/kg, subcutaneous injection; Aladdin Biochemical Technology Co., LTD, Shanghai, China) and then fed in a hypoxic environment (10% O_2_) for 3 weeks, following by room air environment for 2 weeks. Rats in the Con group were fed in room air environment for 5 weeks. After determination of the right ventricular systolic pressure (RVSP) via right heart catheterization under anesthetization, the rats were sacrificed for subsequent experiments.

Experiment 2: Rats were divided into 4 groups: Con, SuHx, SuHx + shNC, SuHx + shTRIM59, with n = 6 for each group. Rats in the Con or SuHx groups received treatment as described previously. Rats in the SuHx + shNC or SuHx + shTRIM59 groups received adeno-associated virus carrying shNC or shTRIM59 (1.2 × 10^8^ v.g, tail vein injection; General Biol Co., Ltd, Chuzhou, China) following by the establishment of SuHx model. After determination of RVSP, the rats were sacrificed for subsequent experiments.

### Cell culture

Human PASMCs were obtained from iCell Bioscience (Shanghai, China) and cultured in iCell primary smooth muscle cell low serum culture system (iCell Bioscience) at 37 °C with 5% CO_2_. For the establishment of a hypoxia-induced cell model, PASMCs were starved in serum-free medium for 3 h, and then cultured in a normoxic condition (21% O_2_, 5% CO_2_) or a hypoxic condition (2% O_2_, 5% CO_2_) for 24 h. Thereafter, the cells were collected for subsequent experiments.

HEK-293 T cells were obtained from ZhongQiaoXinZhou Biotechnology Co., Ltd (Shanghai, China) and cultured in DMEM medium (Servicebio Technology Co., Ltd, Wuhan, China) containing 10% fetal bovine serum (Tianhang Biotechnology Co,. Ltd, Huzhou, China) at 37 °C with 5% CO_2_.

### Infection

PASMCs were infected with adenovirus carrying TRIM59 shRNA, YAP1 shRNA, and TEAD4 shRNA (sequences were listed in Table 1, General Biol Co., Ltd). At 48 h after infection, the cells were starved in serum-free medium for 3 h, cultured in a hypoxic condition for 24 h and then harvested for subsequent experiments. On the other hand, PASMCs were infected with adenovirus carrying TRIM59 over-expression (General Biol Co., Ltd). At 48 h after infection, the cells were harvested for subsequent experiments.

**Table 1:** Sequences of primers, probes and shRNAs

	Gene symbol	Sequences
qPCR primers	Human TRIM59	F: 5′-CCTGCCCTGAACATTAC-3′
		R: 5′-GCTTCCTTATCGCCTTG-3′
	Rat TRIM59	F: 5′-TCAGGCATCTGGTAACTT-3′
		R: 5′-ACATCTGGGTGGTCTTC-3′
	Human YAP1	F: 5′-TGACCCTCGTTTTGCCATGA-3′
		R: 5′-GTTGCTGCTGGTTGGAGTTG-3′
	Human TEAD4	F: 5′- GGCACCATTACCTCCAACG-3′
		R: 5′-TGTAGCGGGCAATCAGC-3′
ChIP primers	TRIM59 promoter (− 1703 ~ − 1692)	F: 5′-TTAAGGAGCTGAATTGGT-3′
		R: 5′-GAGAATCGCTTGAACCC-3′
	TRIM59 promoter (− 1186 ~ − 1174)	F: 5′-GAGGACCAAACCATACCA-3′
		R: 5′-TAGCCACCCAGGAGACAC-3′
Oligonucleotide pull-down probes	WT probe	5′-GGAGTAACAAAATACTTTTTCTTGGAATGTAGTAATCTGTAACCTACCAAATA-3′
	MUT probe	5′-GGAGTAACAAAATACTTTTTGTAGCATTCTTGTAATCTGTAACCTACCAAATA-3′
shRNAs	Human YAP1 shRNA targeted sequence	5′-ATTTAAGAAGTATCTCTGACC-3′
	Human TEAD4 shRNA targeted sequence	5′-TGTTGGTGTTGAGGTCTGCCC-3′
	Human TRIM59 shRNA-1 targeted sequence	5′-TCTAATGTATCATTAAGCTCC-3′
	Human TRIM59 shRNA-2 targeted sequence	5′-ATTAATGTAACTACAACAATG-3′
	Rat TRIM59 shRNA targeted sequence	5′-GTTAACTGTTTAAACAACTTC-3′

### Luciferase reporter assay

Differently truncated fragments of TRIM59 promoter were inserted into PGL3-basic plasmid. The recombinant plasmid was co-transfected into HEK-293 T cells with YAP1 over-expression, TEAD4 over-expression and pRL-TK using Lipofectamine 3000 Reagent (Invitrogen, ThermoFisher Co., Ltd, Waltham, MA, USA) according to the protocol. 48 h later, the luciferase activities were determined with a luciferase activity determination kit (KeyGen Biotechnology Co., LTD, Nanjing, China) according to the manufacturer’s instruction.

### Hematoxylin and eosin (HE) staining

The heart and lung tissues were obtained after indicated treatment. After fixing in 4% paraformaldehyde, the heart and lung tissues were embedded in paraffin and cut into 5-μm slices. The slices were then subjected to deparaffinage and rehydration. Thereafter, the slices were subjected to routine HE staining and observed under a microscope (OLYMPUS, Tokyo, Japan) at 200 × amplification.

### Immunohistochemistry (IHC) staining

After deparaffinage and rehydration, the lung tissue slices from each group were subjected to antigen retrieval in citrate buffer. Endogenous peroxidase was inactivated with 3% H_2_O_2_. After blocked with 1% bovine serum albumin (BSA), the slices were incubated with primary antibodies against TRIM59 (Affinity Bioscience, Changzhou, China; 1: 100) or α-SMA (Proteintech Group, Wuhan, China; 1: 100) at 4 °C overnight. After washing with PBS, the slices were incubated with horseradish peroxidase (HRP)-labeled secondary antibody (ThermoFisher Co., Ltd; 1: 500) at 37 °C for 60 min. After visualization with a DAB substrate kit (Maixin Biotechnology Co., LTD, Fuzhou, China), the slices were counterstained with hematoxylin and observed under a microscope (OLYMPUS) at 100 × , 200 × or 400 × amplifications.

### Immunofluorescence (IF) staining

For IF staining of lung slices, the slices were incubated with antibodies against TRIM59 (Affinity Bioscience; 1: 100), α-SMA (Proteintech Group; 1: 200) or Ki-67 (Affinity Bioscience; 1: 100) at 4 °C overnight after deparaffinage, rehydration, antigen retrieval and blockade with 1% BSA. Thereafter, the slices were incubated with FITC- or Cy3- labeled secondary antibodies (1:200; Abcam, Cambridge, UK; Invitrogen, ThermoFisher Co., Ltd) for 90 min at room temperature, followed by counterstaining with DAPI (Aladdin Biochemical Technology Co., LTD).

For IF staining of cells, the cells were grown on glass coverslips. After fixing in 4% paraformaldehyde, permeabilization in 0.1% tritonX-100 and blockade with 1% BSA, the slices were incubated with antibodies against YAP1 (1: 100; Affinity Bioscience), TEAD4 (1: 100; Proteintech Group), α-SMA (1: 200; Proteintech Group), Calponin (1: 100; Proteintech Group) or Ki-67 (1: 100; Affinity Bioscience) at 4 °C overnight. Thereafter, the slices were incubated with FITC- or Cy3 labeled secondary antibodies for 60 min at room temperature and later counterstained with DAPI. The slices were obtained under a fluorescence microscope (OLYMPUS) at 100 ×, 200 × or 400 × amplification.

### Quantitative real-time PCR (qPCR)

Total RNA was extracted using TRIpure lysis buffer (BioTeke, Beijing, China) according to the instruction’s protocol. After determination of RNA concentration with an ultraviolet spectrophotometer NANO 2000 (ThermoFisher Co., Ltd), The RNA was reversely transcribed into cDNA using BeyoRT II M-MLV (Beyotime Biotechnology Co., Ltd, Shanghaim China) according to the protocol. Thereafter, the mRNA levels of TRIM59, YAP1 and TEAD4 were determined by quantitative real-time PCR (SYBR GREEN method). cDNA served as the template. The primers used were listed in Table 1. The relative mRNA levels of TRIM59, YAP1 and TEAD4 were calculated using 2^−ΔCt^ or 2^−ΔΔCt^ method.

### Western blot

Proteins were extracted using Western lysis buffer (Beyotime Biotechnology Co., Ltd) containing 1 mM PMSF (Beyotime Biotechnology Co., Ltd). After measuring the concentration of proteins, the proteins were separated by 10% SDS-PAGE and then transferred onto polyvinylidene fluoride membranes (Millipore, Bedford, MA, USA). Then membranes were blocked with 5% skim milk following by incubation with primary antibody against TRIM59 (Affinity Bioscience; 1: 1000) at 4 °C overnight. After washing with TBST, the membranes were incubated with HRP-labeled secondary (Beyotime Biotechnology Co., Ltd; 1:5000) at 45 °C for 45 min. Thereafter, the bands of targeted proteins were visualized with an ECL Chemiluminescent kit (Beyotime Biotechnology Co., Ltd).

### Chromatin immunoprecipitation (ChIP)

ChIP was performed with a ChIP assay kit (Wanleibio Technology Co., Ltd, Shenyang, China) according to the protocol. Cells were incubated in 1% formaldehyde for 10 min for crosslink. Later, glycine (0.125 M) was added into cells to stop the crosslink. After washing with PBS, protease inhibitor was added into cells to prevent protein degradation. Chromatin was sheared by sonicator. Thereafter, the fragments were incubated with TRIM59 antibody (Proteintech Group) or IgG (serving as negative control) at 4 °C overnight. Protein A/G beads were added into the mixture to capture the antigen–antibody complex. After immunoprecipitation and elution, RNase was added into the immunoprecipitate and the crosslink was reversed through heating at 65 °C overnight, following by treatment with proteinase K. After DNA recovery, the TRIM59 promoter region was verified by PCR with primers listed in Table 1.

### Oligonucleotide pull-down

Oligonucleotide pull-down was performed with a pull-down kit (BersinBio, Guangzhou, China). First, the biotin-labeled probes (sequences were listed in Table 1) were loaded onto streptavidin-magnetic beads. Later, nuclear extracts were prepared and nucleic acid was removed. Thereafter, the nuclear extracts were mixed with beads loaded with probes in binding buffer containing 5 μL poly(dI⋅dC), 5 μL protease inhibitor, 5 μL DTT, 9 μL EDTA and 4.5 μL EGTA. After incubation for 1 h, the beads were gathered through a magnetic frame. The beads were resuspended and eluted in protein elution buffer. Proteins were then subjected to SDS-PAGE.

### CCK-8

The cells were seeded into 96-well plates (4 × 10^3^ cells/well) in quintuplicate. After indicated treatment, the cells were incubated in a hypoxic condition (2% O_2_, 5% CO_2_) for 24 h. Then 10 μl CCK-8 (KeyGen Biotechnology Co. LTD) was added into each well and incubated at 37 °C for additional 2 h. The absorbance at 450 nm was measured with a microplate reader (BIOTEK, Winooski, VT, USA).

### EdU incorporation assay

EdU incorporation assay was performed with an EdU imaging kit (KeyGen Biotechnology Co., LTD). After indicated treatment, 10 μM EdU staining fluid was added into each well and incubated for additional 2 h. Later, the cells were fixed with 4% paraformaldehyde, permeabilized with 0.5% Triton X-100, and incubated with Click-iT reaction fluid for 30 min. Then the cells were stained with DAPI for 5 min. Images of cells were captured under a fluorescence microscope (OLYMPUS).

### Ubiquitomics

PASMCs were infected with adenovirus carrying TRIM59 over-expression. At 48 h after infection, the cells were harvested to extract proteins for ubiquitomics (QLbio, Beijing, China). After trypsin digestion, ubiquitinated peptides (K-ε-GG) were enriched by immunoprecipitation. Thereafter, the ubiquitinated peptides were subjected to LC–MS/MS analysis. The differently ubiquitylated proteins were analyzed and then subjected to GO analysis and KEGG analysis.

### CO-IP and HPLC–MS/MS

After infection with TRIM59 over-expression, the proteins bound with TRIM59 were identified by LC–MS/MS. Briefly, proteins were extracted using Western and IP cell lysis buffer and immunoprecipitated with anti-flag antibody (Proteintech Group; indicating TRIM59) crosslinked to beads (Beyotime Biotechnology Co., Ltd). After elution, the proteins were identified by HPLC–MS/MS (QLbio).

### Statistical analysis

All data were presented as mean ± SD. Differences among groups were analyzed using Student’s t test or 1-way ANOVA followed by Tukey’s multiple comparison as the post hoc test. p < 0.05 was considered as significant.

## Results

### TRIM59 was highly expressed in PH patients

**Fig. 1:**
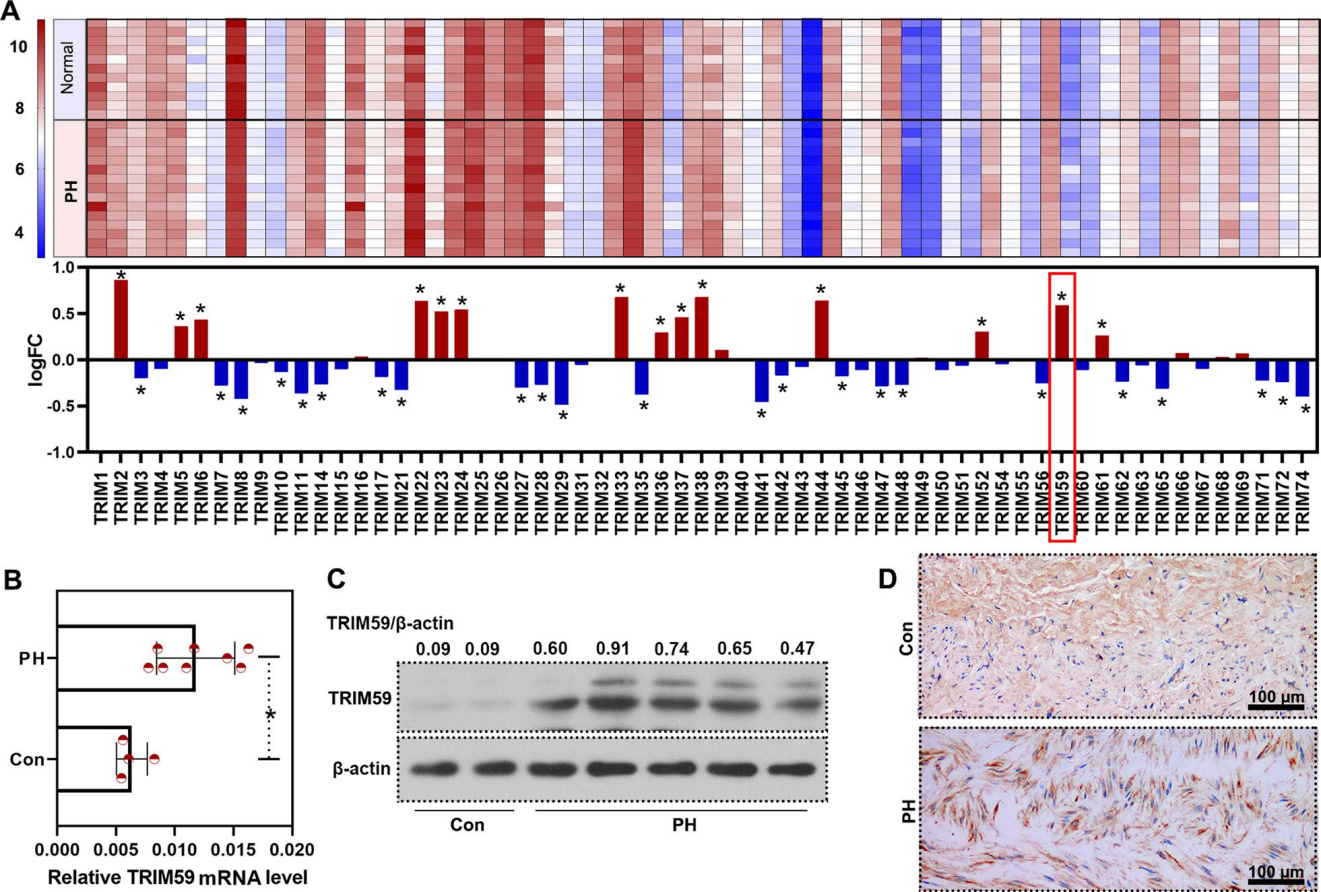
TRIM59 was highly expressed in PH patients. **A** Levels of TRIM family members in the lung tissues of PH patients according to data from GSE113439. **B** qPCR was performed to determine the expression of TRIM59 in the pulmonary arteries from PH patients and controls. **C** The protein level of TRIM59 in the pulmonary arteries of PH patients was detected by western blot. **D** Immunohistochemistry was conducted to detect TRIM59 in the pulmonary arteries of PH patients. Amplification: 200 ×. *p < 0.05

In order to uncover the pathogenesis of PH, we searched GEO database and found GSE113439 which contained the gene expression profile of lung tissues from 15 PH patients and 11 normal controls. Differently expressed genes were analyzed and later we focused on TRIM family. There were 14 up-regulated and 23 down-regulated members of TRIM family (Fig. 1A). Later, we focused on TRIM59, one of the most significantly up-regulated TRIMs. Subsequently, the high expression of TRIM59 in the pulmonary artery tissues from PH patients was verified by qPCR and western blot (Fig. 1B, C). Also, immunohistochemistry staining showed a high TRIM59 level in the pulmonary arteries of PH patients (Fig. 1D), which was consistent with the high expression of TRIM59 revealed by qPCR and western blot in this study, and data from GSE113439. These results demonstrated that TRIM59 was highly expressed in PH patients.

### TRIM59 was highly expressed in the lung tissues of PH rat model and PH cell model

**Fig. 2:**
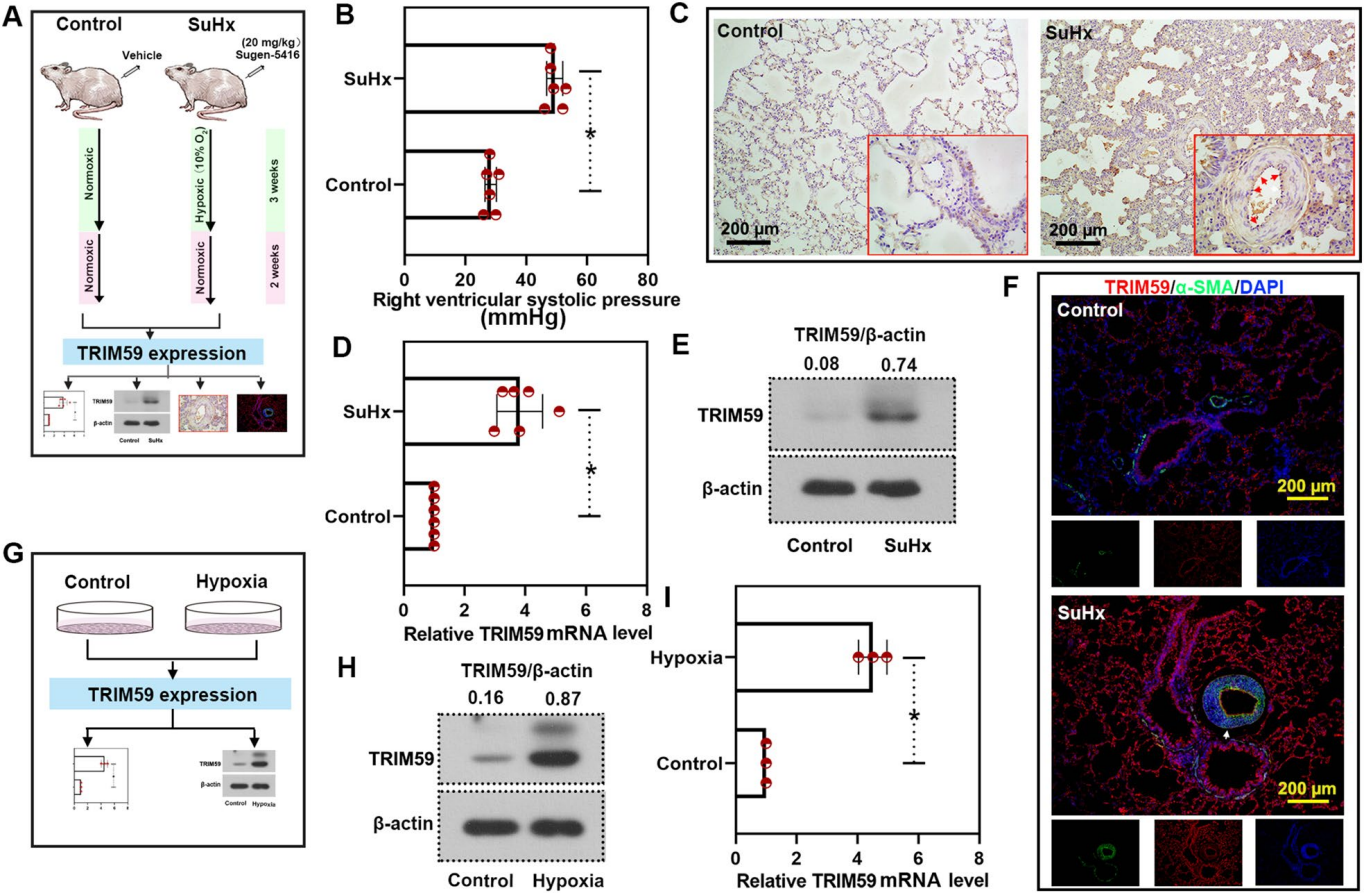
TRIM59 was highly expressed in the lung tissues of rat model and cell model of PH. **A** A rat model of PH was established via a single subcutaneous injection of Sugen-5416 (20 mg/kg) and fed in a hypoxic condition. **B** The right ventricular systolic pressure was measured via right heart catheterization. **C** Immunohistochemistry staining was performed to detect the expression and distribution of TRIM59. Amplification: 100 × and 400 ×. **D** The expression of TRIM59 in the lung tissues of PH rats was determined by qPCR. **E** Western blot was performed to detect the TRIM59 expression in the lung tissues of PH rats. **F** Double immunofluorescence staining was performed to detect the TRIM59 expression and distribution in lung tissues. Amplification: 100 × . **G** Human PASMCs were cultured in hypoxic condition for 24 h. **H** The level of TRIM59 was detected by western blot. **I** qPCR was conducted to determine the expression of TRIM59 in PASMCs under a hypoxic condition. *p < 0.05

In order to determine the expression of TRIM59, a rat model of PH was established via a subcutaneous injection of Sugen-5416 and fed in a hypoxic condition (Fig. 2A). Later, the RVSP was measured in order to verify the success of model establishment (Fig. 2B). Thereafter, the expression and distribution of TRIM59 was determined by immunohistochemistry, immunofluorescence, qPCR and western blot. As shown in the results of immunohistochemistry, high level of TRIM59 was found in the lung tissues of PH rats, especially in the pulmonary artery (Fig. 2C). Later, qPCR and western blot confirmed these results: the lung tissues of PH rats showed higher TRIM59 expression than the control rats (Fig. 2D, E). Moreover, in the lung tissues of PH rats, more TRIM59 was co-located with α-SMA, a marker of smooth muscle cells (Fig. 2F), indicating that PASMCs may contribute to the role of TRIM59 during PH. In addition, human PASMCs were cultured in a hypoxic condition for 24 h to mimic the changes of PASMCs during PH (Fig. 2G). In PASMCs cultured in hypoxic condition, there was an increased TRIM59 expression, as evidenced by qPCR and western blot (Fig. 2G–I). These results were consistent with the results form in vivo experiments. It is demonstrated that TRIM59 may be highly expressed during PH.

### TRIM59 accelerated PASMC proliferation

**Fig. 3:**
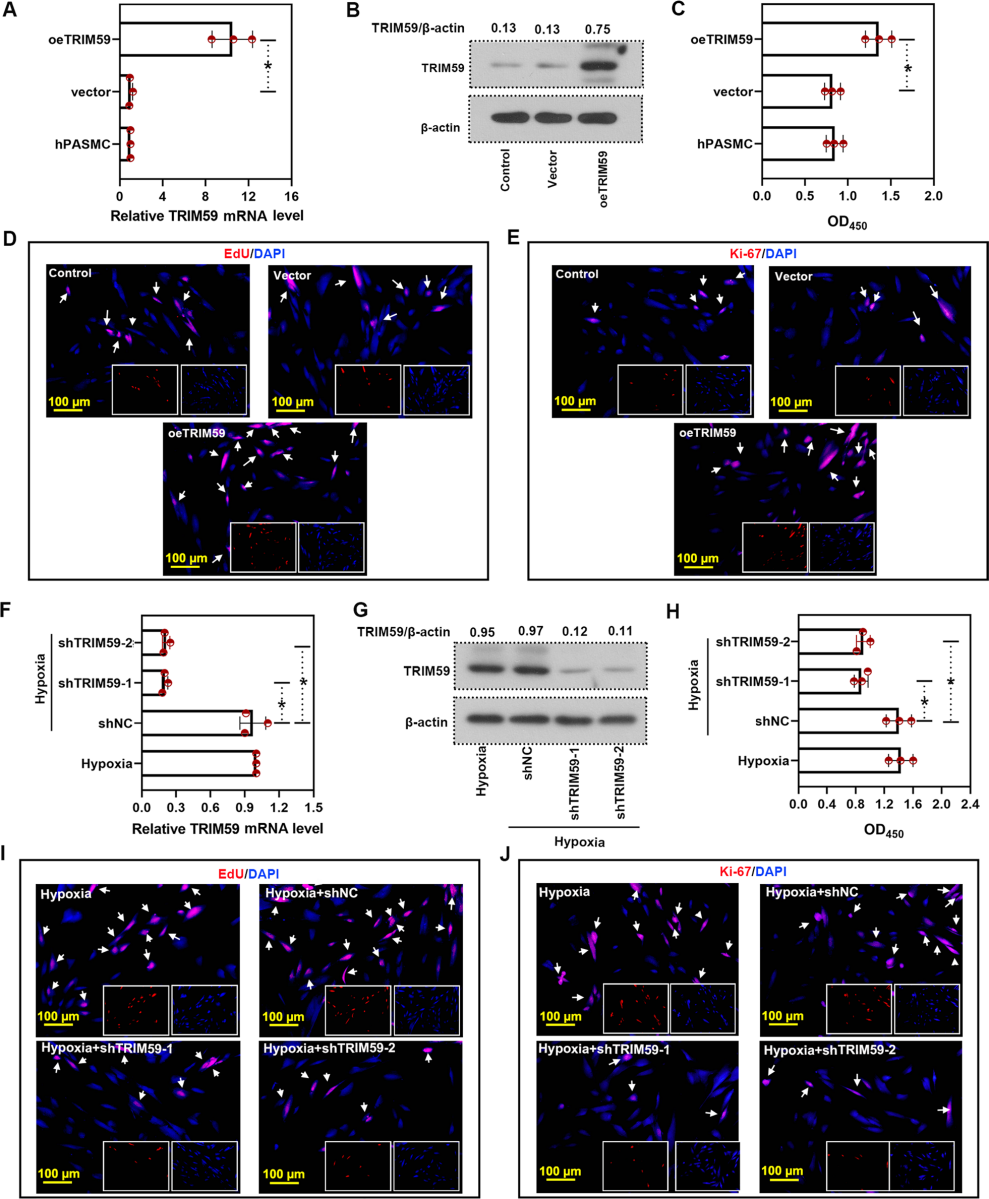
TRIM59 accelerated the proliferation of PASMCs. **A** After TRIM59 over-expression, the mRNA level of TRIM59 was determined by qPCR. **B** Western blot was performed to detect the protein level of TRIM59 after TRIM59 over-expression. **C** After TRIM59 over-expression, the cell viability of PASMCs with TRIM59 over-expression was evaluated by CCK-8 assay. **D** The proliferation of PASMCs was assessed by EdU incorporation assay. Magnification: × 200. White arrows indicated EdU-positive cells. **E** Imunofluorescence staining with Ki-67 antibody was conducted after TRIM59 over-expression. Magnification: × 200. White arrows indicated Ki-67-positive cells. **F** PASMCs with TRIM59 silencing were treated under a hypoxic condition, then the mRNA level of TRIM59 was determined by qPCR. **G** The level of TRIM59 was determined by western blot after indicated treatment. **H** CCK-8 assay was conducted in cells with indicated treatment. **I** EdU incorporation assay was performed to assess the proliferation of PASMC after indicated treatment. Magnification: × 200. White arrows indicated EdU-positive cells. **J** Ki-67 inofluorescence staining was conducted after TRIM59 over-expression. Magnification: × 200. White arrows indicated Ki-67-positive cells. *p < 0.05

As PASMCs contribute to the pathogenesis of PH, we explored the role of TRIM59 in their proliferation. In PASMCs with TRIM59 over-expression, the level of TRIM59 was increased, both at mRNA level and protein level (Fig. 3A, B). After over-expression of TRIM59, the cell viability of PASMCs was also increased (Fig. 3C). Moreover, in cells with TRIM59 over-expression, the level of EdU-positive cells and Ki-67-positive cells was elevated (Fig. 3D, E). In order to explore the role of TRIM59 under hypoxia, cells with TRIM59 silencing were treated under a hypoxic condition. The mRNA and protein levels of TRIM59 were declined after TRIM59 silencing (Fig. 3F, G), along with decreased cell viability (Fig. 3H). Consistently, in cells with TRIM59 silencing, there were less EdU-positive cells and less Ki-67-positive cells (Fig. 3I, J). These results illustrated that TRIM59 accelerated the proliferation of PAMSCs.

### TRIM59 silencing mitigated PH

**Fig. 4:**
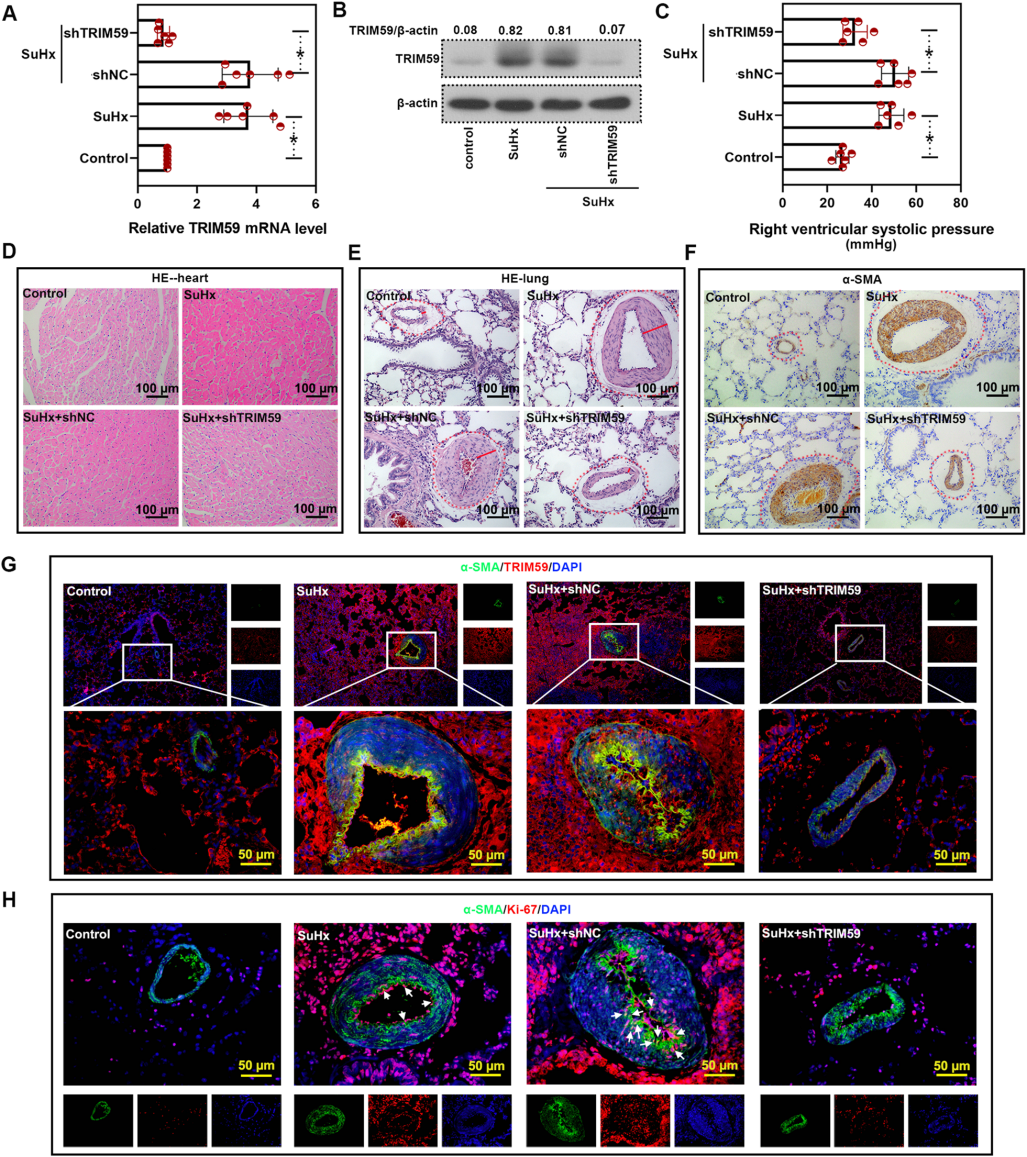
TRIM59 silencing remitted PH. **A** SuHx rat model of PH was established following infection with AAV2 carrying TRIM59 shRNA, then the mRNA level of TRIM59 was determined by qPCR. **B** Western blot was conducted to detect the TRIM59 protein level after TRIM59 silencing in PH rats. **C** After TRIM59 silencing, the right ventricular systolic pressure was measured. **D** HE staining was conducted to evaluate the histopathological changes of heart. Magnification: × 200. **E** HE staining was performed to assess the histopathological changes of lung. Magnification: × 200. **F** Immunohistochemical staining was performed to detect the level of α-SMA. Magnification: × 200. **G** Imunofluorescence staining with TRIM59 and α-SMA was performed. Magnification: 100 ×, 400 ×. **H** The level of α-SMA and Ki-67 was determined by imunofluorescence staining. Magnification: 400 ×. * p < 0.05

In order to explore the role of TRIM59 in PH, rats were infected with AAV2 carrying TRIM59 shRNA to down-regulate TRIM59 in PH rats. The results of qPCR and western blot verified the success of TRIM59 down-regulation (Fig. 4A, B). RVSP was measured to assess the effect of TRIM59 silencing on PH. As before compared, SuHx rats showed a higher RVSP than the control rats. However, after TRIM59 silencing, the RVSP was reduced compared with shNC (Fig. 4C). In addition, the pathological results showed that, compared with control rats, SuHx rats showed disordered and thickened myocardial fibers, while TRIM59 silencing remitted these disordered pathological changes (Fig. 4D). Moreover, compared with control rats, the SuHx rats showed thickened pulmonary artery, while this thickened pulmonary artery was improved after silencing TRIM59 (Fig. 4E). Furthermore, the expression of distribution of α-SMA, a marker of vascular smooth muscle, was also determined by immunohistochemical staining, which results were consistent with the pathological changes. SuHx rats showed more α-SMA and the vascular smooth muscle was thickened. When TRIM59 was silenced, the level of α-SMA was decreased and the vascular smooth muscle was thinned (Fig. 4F). Based on the above results, the expression of TRIM59 in vascular smooth muscle cells was also determined by imunofluorescence double staining with TRIM59 and α-SMA. Compare with control rats, the SuHx rats showed increased TRIM59 level, which was consistent with our previous results. The level of TRIM59 in α-SMA-positive cells was also increased. While TRIM59 silencing decreased both α-SMA-positive cells and the level of TRIM59 in α-SMA-positive cells (Fig. 4G). Furthermore, in cells with TRIM59 silencing, the Ki-67^+^α-SMA^+^ cells were decreased (Fig. 4H), indicating that TRIM59 silencing may suppress the proliferation of vascular smooth muscle cells.

### TRIM59 was transcriptionally regulated by YAP1/TEAD4

**Fig. 5:**
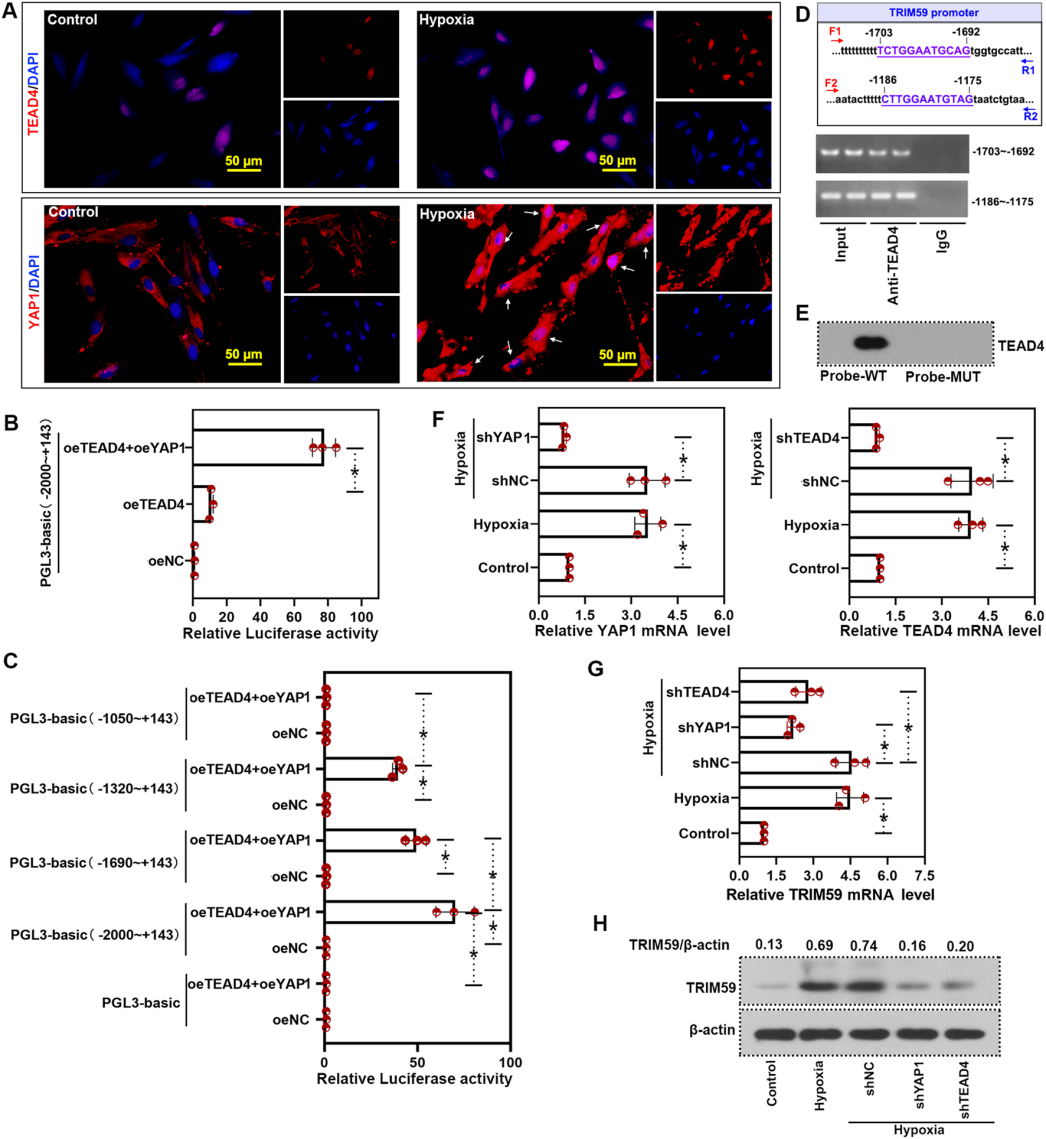
TRIM59 was transcriptionally regulated by YAP1/TEAD4. **A** Immunofluorescence staining was performed to detect YAP1 or TEAD4 expression and distribution during hypoxia. Amplification: 400 ×. **B** The promoter sequence of TRIM59 was inserted into pGL3-basic plasmid. Then the luciferase reporter assay was performed to determine the effect of YAP1 and TEAD4 on the transcription of TRIM59. **C** Differently truncated fragments of TRIM59 promoter were inserted into pGL3-basic plasmid. Then the luciferase reporter assay was performed to determine the effect of YAP1 and TEAD4 on the transcription of TRIM59. **D** ChIP assay was conducted to verify the bind between TEAD4 and TRIM59 promoter. IgG served as the negative control. **E** The binding of TRIM59 promoter and TEAD4 was confirmed by DNA pull down assay. **F** In order to explore the effect of YAP1 and TEAD4 on the transcription of TRIM59, PASMCs were infected with adenovirus carrying YAP1 shRNA or TEAD4 shRNA. Then the levels of YAP1 and TEAD4 were measured by qPCR. **G** After infection, the mRNA level of TRIM59 was determined by qPCR. **H** Western blot was performed to detect the protein level of TRIM59. *p < 0.05

First, the expression and distribution of TEAD4 and YAP1 under hypoxic condition was determined by immunofluorescence staining. TEAD4 mainly located in the nucleus. In PASMCs under hypoxia, there was increased TEAD4 expression. Additionally, YAP1 mainly located in the cytoplasm under normoxic condition and more YAP1 located in the nucleus under hypoxic condition (Fig. 5A). Furthermore, the promoter sequence of TRIM59 was inserted into pGL3-basic plasmid to determine the effect of YAP1 and TEAD4 on the transcription of TRIM59. Compare with negative control (oeNC), TEAD4 over-expression increased the luciferase activity slightly. Moreover, over-expression of TEAD4 together with YAP1 resulted in a significantly higher luciferase activity than TEAD4 over-expression alone (Fig. 5B), indicating that YAP1 reinforced the effect of TEAD4 on TRIM59 transcription. On the other hand, in order to identify the potential binding sites on TRIM59 promoter, differently truncated fragments of TRIM59 promoter were inserted into pGL3-basic plasmid. Compared with oeNC, over-expression TEAD4 with YAP1 increased the luciferase activity under a full-length TRIM59 promoter. However, when the promoter was truncated, the luciferase activity was decreased (Fig. 5C), indicating that there may be binding sites in − 2000 ~ − 1690 and − 1320 ~ − 1050 of TRIM59 promoter. ChIP and DNA pull-down was conducted in order to verify the potential binding sites (Fig. 5D, E). In order to explore the effect of YAP1 and TEAD4 on the transcription of TRIM59, PASMCs were infected with adenovirus carrying YAP1 shRNA or TEAD4 shRNA. Then the levels of YAP1 and TEAD4 were measured by qPCR (Fig. 5F). The mRNA level and protein level of TRIM59 were increased under hypoxia. While silencing YAP1 or TEAD4 resulted in decreased TRIM59 mRNA and protein levels (Fig. 5G, H), indicating the silencing YAP1 or TEAD4 decreased the transcription of TRIM59. It is suggested that TRIM59 was transcriptionally regulated by YAP1/TEAD4.

### TRIM59 modulated protein ubiquitylation

**Fig. 6:**
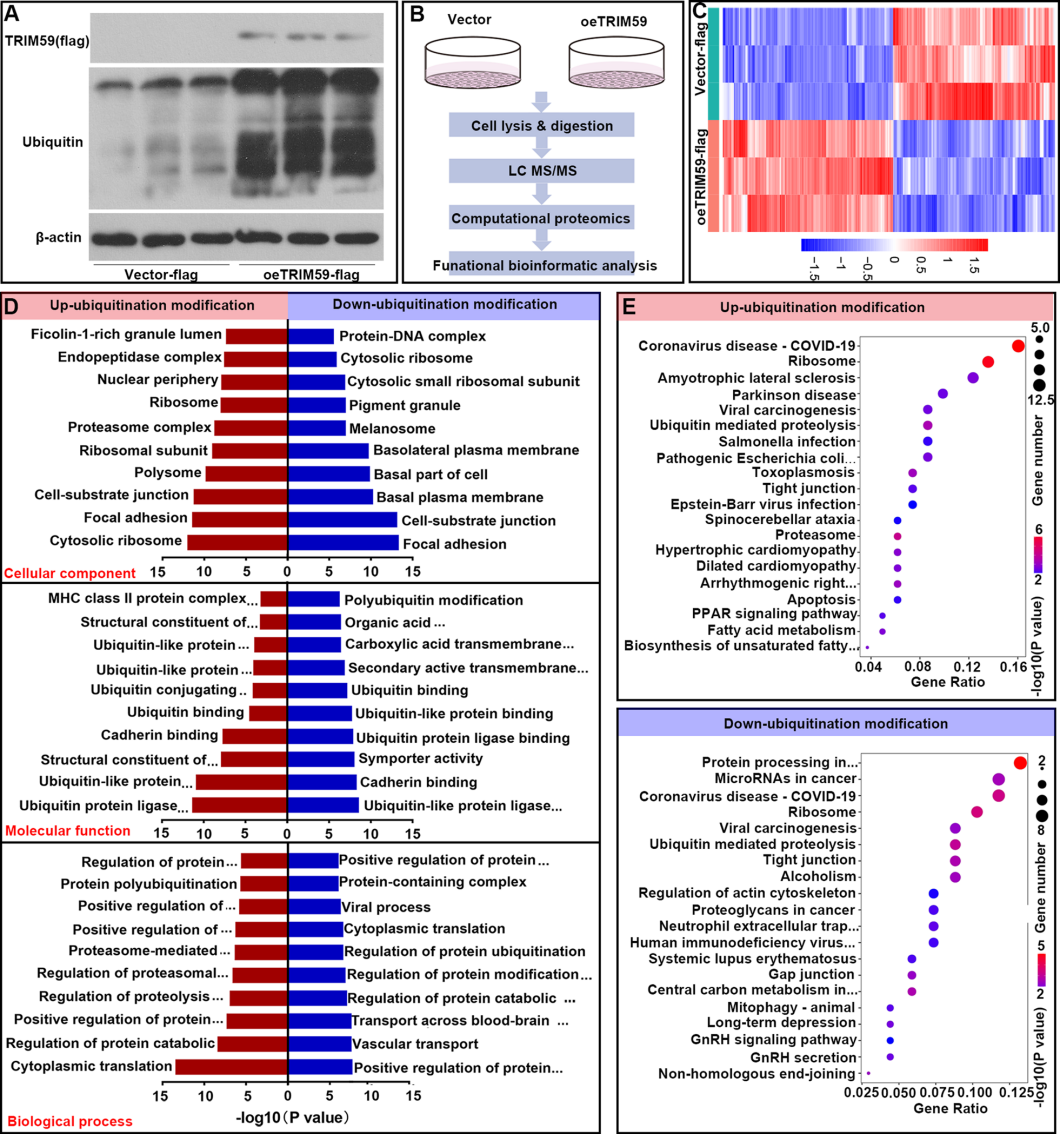
TRIM59 modulated protein ubiquitylation. **A** In PASMCs with TRIM59 over-expression, the level of flag (indicating TRIM59) and total ubiquitylation was detected by western blot. **B** The TRIM59 overexpressed PASMCs were lysed and subjected to protein ubiquitomics. **C** Heatmap of differently ubiquitylated proteins in PASMCs with TRIM59 over-expression. **D** The ubiquitylation-upregulated and ubiquitylation-downregulated proteins were subjected to GO analysis. **E** The differently ubiquitylated proteins were subjected to KEGG analysis

As TRIM59 is an E3 ubiquitin ligating enzyme, the effect of TRIM59 on protein ubiquitylation modulation was detected by protein ubiquitomics. Before protein ubiquitomics, the expression of TRIM59 was verified by western blot. Also, when TRIM59 was upregulated, the level of total ubiquitylation was increased (Fig. 6A). Later, the TRIM59 overexpressed PASMCs were lysed and subjected to protein ubiquitomics as described in Fig. 6B. There were 116 up-ubiquitylated proteins and 103 down-ubiquitylated proteins (Fig. 6C, Table 2). These differently ubiquitylated proteins were then subjected to GO analysis. For the up-ubiquitylated proteins, the most common cellular component (CC) items were cytosolic ribosome, focal adhesion and cell-substrate junction; the most common molecular function (MF) items were ubiquitin protein ligase binding, ubiquitin-like protein ligase binding and structural constituent of ribosome; the most common biological process (BP) items were regulation of protein catabolic process and positive regulation of protein catabolic process. For the down-ubiquitylated proteins, the most common CC items were focal adhesion, cell-substrate junction and basal plasma membrane; the most common MF items were ubiquitin-like protein ligase binding, cadherin binding and symporter activity; the most common BP items were positive regulation of protein catabolic process, vascular transport and transport across blood–brain barrier (Fig. 6D). In addition, these differently ubiquitylated proteins were also subjected to KEGG assay. The up-ubiquitylated proteins were mainly involved in coronavirus disease-COVID-19, ribosome, proteasome, ubiquitin mediated proteolysis and toxoplasmosisl; while the down-ubiquitylated proteins were mainly implicated in protein processing in endoplasmic reticulum, ribosome, coronavirus disease-COVID-19, ubiquitin mediated proteolysis and central carbon metabolism in cancer (Fig. 6E). These data indicated that TRIM59 modulated the ubiquitylation of proteins. These differently ubiquitylated proteins were implicated in various cellular components, molecular functions, biological processes and pathways.

**Table 2:** Up-ubiquitination and down-ubiquitination modified by TRIM59

Gene symbol	Ubiquitination site	Fold change	Description
Up-ubiquitination modification			
ACSL4	K702	6.02	Long-chain-fatty-acid–CoA ligase 4
	K401	28.94	
ACTB	K328	4.97	Actin, cytoplasmic 1
AHCY	K188	2.24	Adenosylhomocysteinase
AKAP2	K448	5.23	A-kinase anchor protein 2
ALDOA	K42	2.33	Fructose-bisphosphate aldolase A
ANKRD13A	K247	11.82	Ankyrin repeat domain-containing protein 13A
	K219	7.93	
APBB1	K48	5.79	Amyloid beta precursor protein binding family B member 1
CAVIN1	K170	17.71	Caveolae-associated protein 1
	K122	9.64	
CCDC50	K129	1.43	Coiled-coil domain-containing protein 50
CDC20	K490	6.30	Cell division cycle protein 20 homolog
CHIC1	K176	5.05	Cysteine-rich hydrophobic domain-containing protein 1
CLIC4	K199	1.94	Chloride intracellular channel protein 4
CRIM1	K978	3.64	Cysteine-rich motor neuron 1 protein
CSDE1	K91	3.37	Cold shock domain-containing protein E1
CUEDC1	K318	2.31	CUE domain-containing protein 1
CYB5R3	K163	3.92	NADH-cytochrome b5 reductase 3
CYP1B1	K454	3.57	Cytochrome P450 1B1
DAG1	K793	4.68	Dystroglycan 1
	K782	31.67	
DCBLD2	K560	1.70	Discoidin, CUB and LCCL domain-containing protein 2
EEF2	K272	8.43	Elongation factor 2
	K239	1.66	
EHMT1	K827	3.33	Histone-lysine N-methyltransferase EHMT1
EPS15L1	K531	3.80	Epidermal growth factor receptor substrate 15-like 1
FADS2	K409	3.58	Acyl-CoA 6-desaturase
	K28	22.75	
	K108	5.67	
GABARAP	K13	4.36	Gamma-aminobutyric acid receptor-associated protein
GAPDH	K194	3.77	Glyceraldehyde-3-phosphate dehydrogenase
GJA1	K303	16.95	Gap junction alpha-1 protein
	K258	5.67	
	K128	7.89	
GNAI2	K46	2.26	Guanine nucleotide-binding protein G(i) subunit alpha-2
GPRC5A	K348	4.93	Retinoic acid-induced protein 3
GRB2	K109	1.92	Growth factor receptor-bound protein 2
H1-5	K93	8.22	Histone H1.5
	K88	2.86	
HMGCR	K248	2.50	3-hydroxy-3-methylglutaryl-coenzyme A reductase
HNRNPA1	K350	12.44	Heterogeneous nuclear ribonucleoprotein A1
HNRNPK	K198	2.54	Heterogeneous nuclear ribonucleoprotein K
HSP90AA1	K443	6.15	Heat shock protein HSP 90-alpha
	K362	19.99	
HSPA8	K71	2.91	Heat shock cognate 71 kDa protein
IFNGR1	K285	4.02	Interferon gamma receptor 1
ITGB1	K794	7.85	Integrin beta-1
JAK1	K504	12.53	Tyrosine-protein kinase JAK1
	K227	8.53	
LMNA	K270	11.70	Prelamin-A/C
LMNB1	K389	4.70	Lamin-B1
	K102	6.05	
LSS	K432	1.97	Lanosterol synthase
MAGED1	K656	4.57	Melanoma-associated antigen D1
MCL1	K40	2.22	Induced myeloid leukemia cell differentiation protein Mcl-1
MMP15	K662	9.19	Matrix metalloproteinase-15
MTA2	K611	4.24	Metastasis-associated protein MTA2
MYH10	K689	2.02	Myosin-10
MYRF	K749	3.47	Myelin regulatory factor
NCKAP1	K12	6.03	Nck-associated protein 1
NDFIP2	K193	2.11	NEDD4 family-interacting protein 2
NEDD4	K874	17.30	E3 ubiquitin-protein ligase NEDD4
NME2	K12	7.44	Nucleoside diphosphate kinase B
	K100	1.69	
NONO	K198	2.76	Non-POU domain-containing octamer-binding protein
OTUB1	K84	1.42	Ubiquitin thioesterase OTUB1
PABPC4	K188	3.04	Polyadenylate-binding protein 4
PI4K2A	K240	5.04	Phosphatidylinositol 4-kinase type 2-alpha
PKM	K270	1.89	Pyruvate kinase PKM
PLCXD1	K227	2.31	PI-PLC X domain-containing protein 1
PPP2R1B	K200	6.95	Serine/threonine-protein phosphatase 2A 65 kDa regulatory subunit A beta isoform
PRKAR1A	K261	3.80	cAMP-dependent protein kinase type I-alpha regulatory subunit
PSMA7	K115	3.09	Proteasome subunit alpha type-7
PSMB3	K77	7.85	Proteasome subunit beta type-3
PSMC3	K245	6.84	26S proteasome regulatory subunit 6A
PSMC5	K94	1.37	26S proteasome regulatory subunit 8
PSMD2	K66	2.17	26S proteasome non-ATPase regulatory subunit 2
PTBP1	K508	4.84	Polypyrimidine tract-binding protein 1
RAB5C	K117	17.55	Ras-related protein Rab-5C
RACGAP1	K292	1.95	Rac GTPase-activating protein 1
RACK1	K106	3.77	Receptor of activated protein C kinase 1
RAD18	K218	2.00	E3 ubiquitin-protein ligase RAD18
RDX	K79	2.07	Radixin
RNF114	K126	4.31	E3 ubiquitin-protein ligase RNF114
RNF167	K272	1.61	E3 ubiquitin-protein ligase RNF167
RPL12	K61	4.28	60S ribosomal protein L12
RPL24	K93	2.45	60S ribosomal protein L24
	K27	3.73	
RPL3	K286	3.50	60S ribosomal protein L3
RPL30	K26	5.15	60S ribosomal protein L30
RPL31	K70	4.31	60S ribosomal protein L31
RPL6	K262	2.49	60S ribosomal protein L6
RPL7A	K48	2.17	60S ribosomal protein L7a
	K245	4.07	
RPS17	K19	11.59	40S ribosomal protein S17
RPS27A	K6	2.12	Ubiquitin-40S ribosomal protein S27a
RPS3	K141	2.02	40S ribosomal protein S3
RPSA	K89	5.62	40S ribosomal protein SA
RRM1	K376	2.47	Ribonucleoside-diphosphate reductase large subunit
SCD	K341	6.02	Stearoyl-CoA desaturase
SCP2	K438	9.43	Sterol carrier protein 2
SFPQ	K421	2.20	Splicing factor, proline- and glutamine-rich
SGTA	K200	8.44	Small glutamine-rich tetratricopeptide repeat-containing protein alpha
	K160	9.16	
SLC20A1	K389	1.78	Sodium-dependent phosphate transporter 1
SMARCA5	K929	6.41	SWI/SNF-related matrix-associated actin-dependent regulator of chromatin subfamily A member 5
SPART	K481	2.37	Spartin
SPTLC1	K197	4.47	Serine palmitoyltransferase 1
SUV39H1	K138	5.91	Histone-lysine n-methyltransferase SUV39H1
SYNCRIP	K363	17.81	Heterogeneous nuclear ribonucleoprotein Q
TCAF1	K817	11.59	TRPM8 channel-associated factor 1
TMUB1	K129	2.73	Transmembrane and ubiquitin-like domain-containing protein 1
	K108	4.00	
TNFRSF1A	K242	7.92	Tumor necrosis factor receptor superfamily member 1A
TPD52L2	K108	1.74	Tumor protein D54
TPM1	K37	1.51	Tropomyosin alpha-1 chain
TPR	K290	2.67	Nucleoprotein TPR
UBE2L3	K73	2.11	Ubiquitin-conjugating enzyme E2 L3
UBE2N	K82	8.56	Ubiquitin-conjugating enzyme E2 N
UBE2O	K132	5.16	(E3-independent) E2 ubiquitin-conjugating enzyme
UBE2T	K91	2.10	Ubiquitin-conjugating enzyme E2 T
	K48	2.74	
UBE3A	K330	1.88	Ubiquitin-protein ligase E3A
UBTD2	K68	2.88	Ubiquitin domain-containing protein 2
UCK2	K78	7.42	Uridine-cytidine kinase 2
USP14	K441	8.10	Ubiquitin carboxyl-terminal hydrolase 14
USP25	K444	5.44	Ubiquitin carboxyl-terminal hydrolase 25
	K201	2.12	
USP5	K423	5.82	Ubiquitin carboxyl-terminal hydrolase 5
VAMP3	K35	6.18	Vesicle-associated membrane protein 3
VCP	K658	15.10	Transitional endoplasmic reticulum ATPase
	K236	14.68	
VIM	K445	4.05	Vimentin
WIZ	K1597	5.95	Protein Wiz
YWHAZ	K49	3.87	14-3-3 protein zeta/delta
ZFYVE16	K1257	46.92	Zinc finger FYVE domain-containing protein 16
Down-ubiquitination modification			
SLC4A7	K548	0.17	Sodium bicarbonate cotransporter 3
	K31	0.39	
RNF114	K161	0.14	E3 ubiquitin-protein ligase RNF114
UBQLN1	K47	0.36	Ubiquilin-1
TNFRSF10D	K241	0.29	Tumor necrosis factor receptor superfamily member 10D
UBAP1	K177	0.56	Ubiquitin-associated protein 1
FANCI	K523	0.39	Fanconi anemia group I protein
ACBD3	K386	0.43	Golgi resident protein GCP60
FAM234A	K15	0.61	Protein FAM234A
RNF5	K93	0.36	E3 ubiquitin-protein ligase RNF5
SLC29A1	K263	0.56	Equilibrative nucleoside transporter 1
	K255	0.63	
	K249	0.54	
PKP4	K518	0.44	Plakophilin-4
UIMC1	K31	0.19	BRCA1-A complex subunit RAP80
DCUN1D1	K149	0.49	DCN1-like protein 1
RNF185	K105	0.25	E3 ubiquitin-protein ligase RNF185
STAM	K171	0.64	Signal transducing adapter molecule 1
MICAL1	K1036	0.39	[F-actin]-monooxygenase MICAL1
UBA3	K147	0.27	NEDD8-activating enzyme E1 catalytic subunit
SLC43A3	K264	0.25	Equilibrative nucleobase transporter 1
	K255	0.19	
	K249	0.31	
KTN1	K256	0.42	Kinectin
H2AC25	K96	0.38	Histone H2A type 3
SV2A	K143	0.45	Synaptic vesicle glycoprotein 2A
TUBA1A	K60	0.24	Tubulin alpha-1A chain
ZDHHC20	K96	0.24	Palmitoyltransferase ZDHHC20
H2BC21	K109	0.56	Histone H2B type 2-E
H2AC20	K119	0.57	Histone H2A type 2-C
VAMP3	K42	0.38	Vesicle-associated membrane protein 3
UBE2V2	K72	0.45	Ubiquitin-conjugating enzyme E2 variant 2
SLC1A5	K10	0.46	Neutral amino acid transporter B(0)
ABRAXAS2	K229	0.35	BRISC complex subunit Abraxas 2
EIF3A	K420	0.36	Eukaryotic translation initiation factor 3 subunit A
ADAM9	K781	0.51	Disintegrin and metalloproteinase domain-containing protein 9
TRIM28	K750	0.04	Transcription intermediary factor 1-beta
CALCOCO2	K339	0.25	Calcium-binding and coiled-coil domain-containing protein 2
AHNAK	K4945	0.50	Neuroblast differentiation-associated protein AHNAK
SLC20A2	K262	0.37	Sodium-dependent phosphate transporter 2
GFPT1	K48	0.20	Glutamine–fructose-6-phosphate aminotransferase 1
UBE3A	K323	0.55	Ubiquitin-protein ligase E3A
UBXN1	K83	0.49	UBX domain-containing protein 1
RPL19	K186	0.41	60S ribosomal protein L19
BASP1	K38	0.33	Brain acid soluble protein 1
PRKDC	K810	0.36	DNA-dependent protein kinase catalytic subunit
UBE2L3	K96	0.40	Ubiquitin-conjugating enzyme E2 L3
	K82	0.21	
	K138	0.67	
	K131	0.46	
YBX1	K137	0.69	Y-box-binding protein 1
YWHAZ	K9	0.60	14-3-3 protein zeta/delta
RPS27A	K27	0.75	Ubiquitin-40S ribosomal protein S27a
HNRNPK	K422	0.10	Heterogeneous nuclear ribonucleoprotein K
SUMO2	K33	0.42	Small ubiquitin-related modifier 2
UBE2M	K92	0.68	NEDD8-conjugating enzyme Ubc12
RPS20	K8	0.56	40S ribosomal protein S20
VCP	K251	0.27	Transitional endoplasmic reticulum ATPase
	K231	0.62	
	K109	0.56	
SLC12A2	K976	0.30	Solute carrier family 12 member 2
RAD23B	K51	0.46	UV excision repair protein RAD23 homolog B
ATXN3	K200	0.72	Ataxin-3
SLC16A1	K473	0.17	Monocarboxylate transporter 1
	K224	0.41	
CAPZB	K267	0.25	F-actin-capping protein subunit beta
NEDD4	K882	0.47	E3 ubiquitin-protein ligase NEDD4
RPS10	K138	0.38	40S ribosomal protein S10
	K139	0.50	
RPS9	K155	0.63	40S ribosomal protein S9
USP5	K360	0.72	Ubiquitin carboxyl-terminal hydrolase 5
SLC1A4	K528	0.14	Neutral amino acid transporter A
	K501	0.05	
	K493	0.14	
	K484	0.17	
	K3	0.29	
PEX19	K52	0.34	Peroxisomal biogenesis factor 19
BSG	K366	0.22	Basigin
RDX	K526	0.48	Radixin
SLC6A6	K19	0.31	Sodium- and chloride-dependent taurine transporter
GNA11	K107	0.44	Guanine nucleotide-binding protein subunit alpha-11
MARCKS	K11	0.20	Myristoylated alanine-rich C-kinase substrate
MSN	K165	0.28	Moesin
	K162	0.31	
	K139	0.47	
ATP2B4	K735	0.34	Plasma membrane calcium-transporting ATPase 4
CFL1	K144	0.48	Cofilin-1
RPS3	K214	0.42	40S ribosomal protein S3
PAM	K901	0.62	Peptidyl-glycine alpha-amidating monooxygenase
TCP1	K541	0.43	T-complex protein 1 subunit alpha
PRKCA	K316	0.54	Protein kinase C alpha type
STMN1	K52	0.35	Stathmin
H1-3	K65	0.45	Histone H1.3
	K35	0.52	
H1-5	K49	0.71	Histone H1.5
	K37	0.43	
H2AX	K128	0.45	Histone H2AX
RPS2	K58	0.37	40S ribosomal protein S2
CD46	K375	0.49	Membrane cofactor protein
EZR	K139	0.65	Ezrin
XRCC5	K195	0.07	X-ray repair cross-complementing protein 5
PCNA	K164	0.52	Proliferating cell nuclear antigen
HSPA8	K524	0.57	Heat shock cognate 71 kDa protein
CNP	K261	0.22	2′,3′-cyclic-nucleotide 3′-phosphodiesterase
VIM	K188	0.34	Vimentin
SLC3A2	K147	0.52	4F2 cell-surface antigen heavy chain
LDHB	K308	0.20	l-lactate dehydrogenase B chain
RPN1	K538	0.42	Dolichyl-diphosphooligosaccharide–protein glycosyltransferase subunit 1
HLA-A	K340	0.39	HLA class I histocompatibility antigen, A alpha chain
	K364	0.09	
	K200	0.37	
LMNA	K97	0.26	Prelamin-A/C
	K180	0.51	
NRAS	K128	0.31	GTPase NRas
TDP2	K82	0.35	Tyrosyl-DNA phosphodiesterase 2
MPZL1	K213	0.74	Myelin protein zero-like protein 1
UBXN7	K84	0.03	UBX domain-containing protein 7
SLC16A7	K463	0.23	Monocarboxylate transporter 2
NUDT21	K29	0.55	Cleavage and polyadenylation specificity factor subunit 5
EPB41L2	K374	0.58	Band 4.1-like protein 2
	K144	0.17	
	K114	0.34	
SLC16A3	K448	0.49	Monocarboxylate transporter 4
	K431	0.44	
	K428	0.39	
SLC16A4	K244	0.18	Monocarboxylate transporter 5
SCAMP1	K89	0.55	Secretory carrier-associated membrane protein 1
SCAMP3	K101	0.00	Secretory carrier-associated membrane protein 3
EEF2K	K341	0.18	Eukaryotic elongation factor 2 kinase
CLIC1	K119	0.31	Chloride intracellular channel protein 1

### Direct targets of TRIM59

**Fig. 7:**
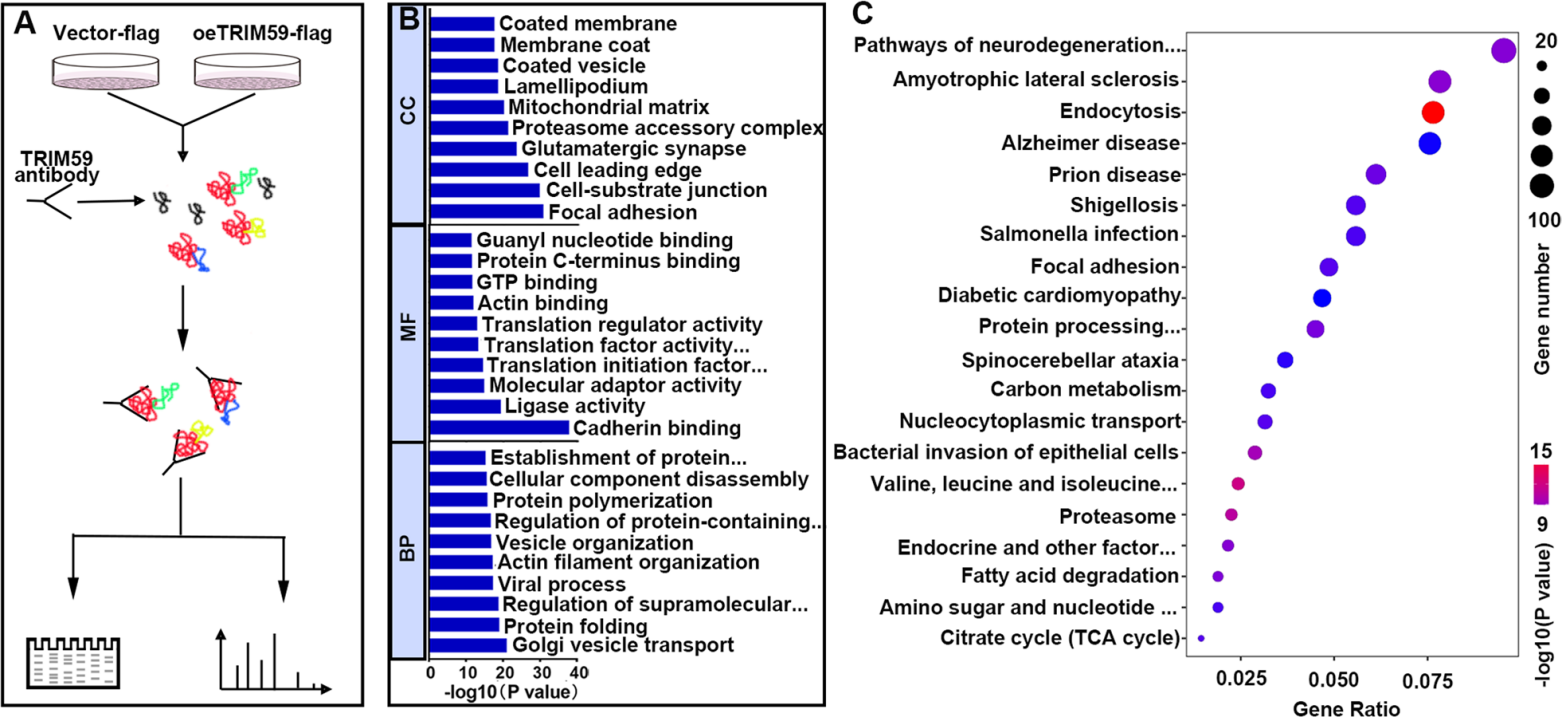
Binding proteins of TRIM59. **A** CoIP was performed with flag antibody (indicating TRIM59) to detect the proteins binding to TRIM59. **B** CoIP binding proteins were subjected to GO analysis. **C** CoIP binding proteins were subjected to KEGG analysis

In order to identify direct targets of TRIM59, CO-IP companied with LC–MS/MS was conducted (Fig. 7A). Later, the CO-IP products were subjected to LC–MS/MS and identified 1924 binding proteins. The CO-IP binding proteins were subjected to GO and KEGG assay. The most common CC items were focal adhesion, cell-substrate junction and cell leading edge; the most common MF items were cadherin binding, ligase activity and molecular adaptor activity; the most common BP items were golgi vesicle transport, protein folding and regulation of supramolecular fiber organization (Fig. 7B); the most implicated pathways were endocytosis, valine, leucine and isoleucine degradation, proteasome, bacterial invasion of epithelial cells and amyotrophic lateral sclerosis (Fig. 7C).

**Fig. 8:**
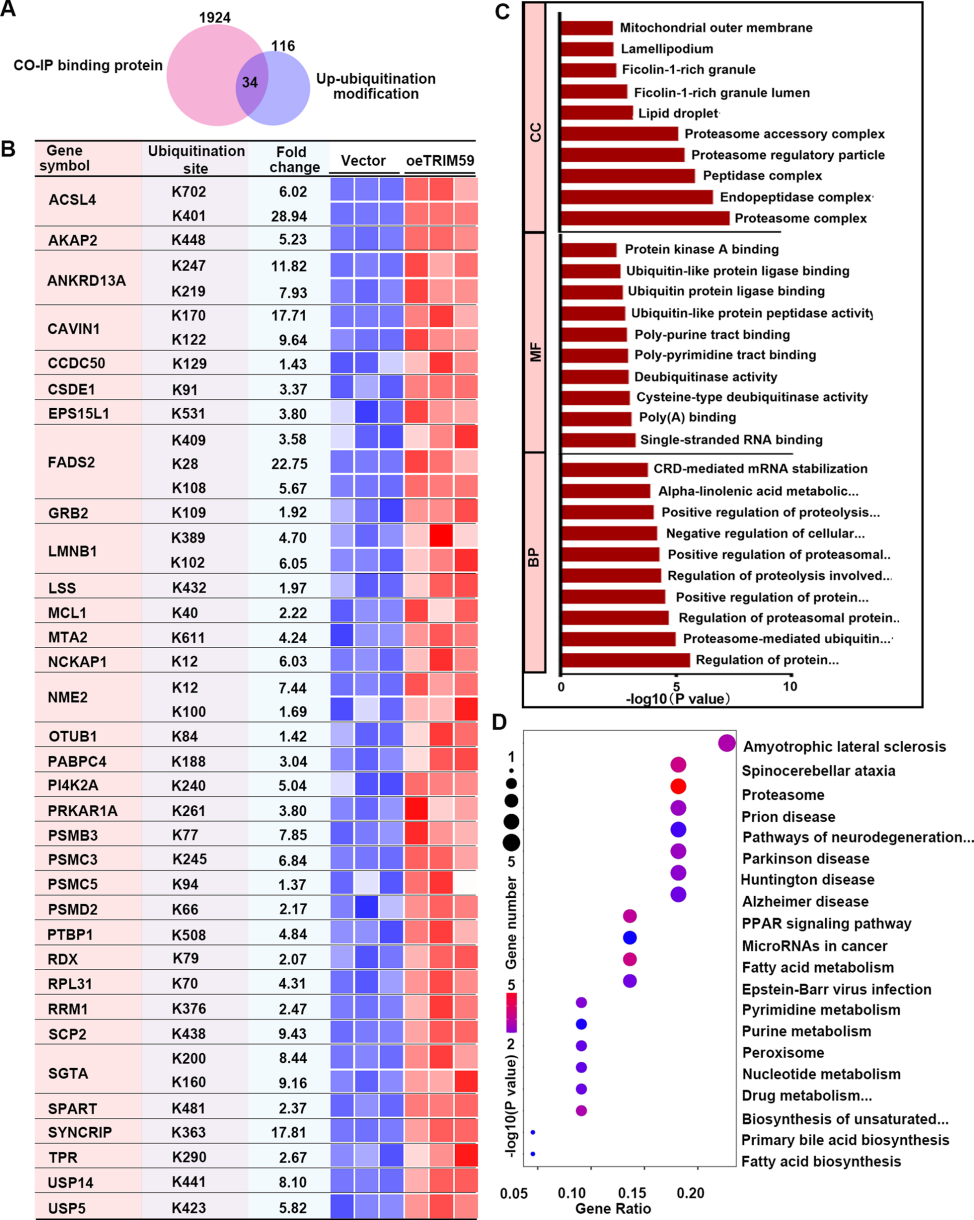
Direct targets of TRIM59. **A** Venn diagram of CoIP binding proteins and up- ubiquitylated proteins. **B** Direct targets of TRIM59 identified by protein ubiquitomics and CoIP-LC–MS/MS. **C** TRIM59 direct targets were subjected to GO analysis. **D** TRIM59 direct targets were subjected to KEGG analysis

Comprehensive analysis up-ubiquitylated proteins and CO-IP binding proteins, we identified 34 direct targets of TRIM59 (Fig. 8A, B), which were both bound with TRIM59 and up-ubiquitylated by TRIM59. For these direct target proteins, the most common CC items were proteasome complex, endopeptidase complex and peptidase complex; the most common MF items were single-stranded RNA binding, poly(A) binding and cysteine-type deubiquitinase activity; the most common BP items were regulation of protein catabolic process, proteasome-mediated ubiquitin-dependent protein catabolic process, and regulation of proteasomal protein catabolic process (Fig. 8C). These direct target proteins were mainly involved in pathways such as proteasome, fatty acid metabolism, spinocerebellar ataxia, PPAR signaling pathway, and amyotrophic lateral sclerosis. These direct target proteins were associated with various cellular components, molecular functions, biological processes and pathways (Fig. 8D).

## Discussion

Recent PH treatment predominantly targets vasoactive mediators. Although, along with the development of nanomaterials, novel drug delivery systems are developed in order to reduce the side effects [18], these drugs are still with their limitations: no effects on pulmonary vascular remodeling. Thus, exploring the molecular mechanisms of PH pulmonary vascular remodeling is important to the development of novel PH treatment. In the present study, we uncovered the role of TRIM59 in the pathogenesis of PH. TRIM59, which was highly expressed during PH, promoted the proliferation of PASMCs. Meanwhile, silencing TRIM59 mitigated PH via modulating the ubiquitylation of proteins. It is indicated that TRIM59 may be involved in the molecular mechanisms of PASMC excessive proliferation and contribute to the pathogenesis of PH.

According to a GEO dataset GSE113439, TRIM59 was highly expressed in the lung tissues from PH patients (by array). Consistently, its expression in the pulmonary arteries from PH patients was verified at both mRNA level and protein level. We also confirmed the high TRIM59 expression in a SuHx rat model of PH and a hypoxia-induced cell model. These data indicated that TRIM59 may relate to the pathogenesis of PH. Other genes differently expressed in PH may also relate to PH, which may be explored in our future study.

TRIM59 participates in the development of pulmonary diseases. It was reported that TRIM59 knockout aggravated the pulmonary injury induced by sepsis [19]. While, TRIM59 promoted the chemoresistance of lung cancer [20]. In the present study, we explored the role of TRIM59 in PH. Silencing TRIM59 mitigated the heart and lung injuries during PH, especially pulmonary artery remodeling, indicating a protective role of TRIM59 silencing in PH. Furthermore, we also found that TRIM59 silencing suppressed the proliferation of α-SMA-positive PASMC in vivo. As we know, during PH, PASMCs undergo a complicated signal program, which leads to the loss of growing suppression and persistent activation of signals associated with pro-proliferation and pro-survival. Now, it is widely considered that excessive PASMC proliferation is one of the prominent hallmarks of PH [21] and contributes to vascular remodeling. Our in vitro study also revealed that TRIM59 accelerated the proliferation of PASMCs. Meanwhile, silencing TRIM59 abolished hypoxia-induced proliferation of PASMCs. It is indicated that the accelerating proliferation role of TRIM59 may contribute to its function during PH. Interestingly, TRIM59 was also reported to repress the production of NO [19], a kind of vasodilator, indicating that TRIM59 may also suppress vasodilatation during PH, which needs further exploration.

We wondered why TRIM59 was highly expressed during PH. In the promoter sequence of TRIM59, we found the potential binding sequences for YAP1/TEAD4 complex. YAP1, as a transcriptional co-activator, contributes to the proliferation of cells through modulating the transcription of growth-related genes [22, 23]. Up-regulation of YAP1 accelerated the proliferation of PASMCs and remodeling of pulmonary artery [24]. Members of The TEAD family are the main transcription factors mediating the function of YAP1 [25, 26]. In our study, we found that in PASMCs cultured in a hypoxic condition, the nuclear YAP1 and TEAD4 levels were increased, indicating their highly activated status. Consistently, Wang et al. also showed an activated YAP1 in MCT-induced PH rat models [15]. Later, we confirmed that YAP1/TEAD4 promoted the transcription of TRIM59, which may contribute to the high level of TRIM59 during PH. Interestingly, we also found that TEAD4 alone could promote TRIM59 transcription (although slightly weak), but YAP1 enhanced this effect of TEAD4. Moreover, increasing evidence indicates that TEAD4 may also perform its functions through a YAP1-independent manner.

E3 ubiquitin ligases play critical roles in determining the specificity of substrates, recognizing the degradation signals and catalyzing the transferation of ubiquitin to substrates [27]. Their aberrance is associated with a variety of cell processes and diseases, including PH [15, 28]. We found that TRIM59, which was highly expressed during PH, heightened the pan-ubiquitination, which may contribute to its role during PH. As TRIM59 is one of the E3 ubiquitin ligases, we performed a protein ubiquitomics in order to explore its role in the protein ubiquitination. There were 137 up-ubiquitylated sites. Except items associated with ubiquitination modification and catalytic degradation, these up-ubiquitylated proteins were associated with histone methylation and regulation of translation, indicating that TRIM59 may modulate the transcription process indirectly. Interestingly, among these up-ubiquitylated proteins, most proteins showed only one ubiquitylated site, while ACSL4, ANKRD13A, CAVIN1, DAG1, EEF2, HSP90AA1, JAK1, LMNB1, NME2, RPL24, RPL7A, RPL7A, UBE2T, USP25, and VCP showed 2 ubiquitylated sites and FADS2 and GJA1 showed 3 ubiquitylated sites. There were also 103 down-ubiquitylated proteins, which may due to an indirect function of TRIM59.

E3 ubiquitin ligases play their roles mainly through binding with their substrates specifically. Thus, we also performed a CO-IP and then used LC–MS/MS to identify the binding proteins of TRIM59. After combined analysis of binding proteins and ubiquitylated proteins, we identified 34 proteins as the direct substrates of TRIM59. Among these direct targets of TRIM59, the suppression of CAVIN1 aggravated pulmonary artery endothelium injury and apoptosis [29], and suppressing MCL-1 promoted PASMC apoptosis exposed to hypoxia [30]. Thus, we thought these two proteins were the direct targets of TRIM59. However, among these direct targets of TRIM59 identified in this study, PTBP1 silence suppresses the phenotypic transition of PASMCs during PH [31], USP14 promoted the proliferation and migration of vascular smooth muscle cells via mTOR/P70S6K signaling pathway [32], and TPR promoted cellular hypertrophy of vascular smooth muscle cells [33] and enhanced oxidative stress and inflammation [34]. Also, downregulation of GRB2 was reported to mediate the protective effect of dioscin on PH [35]. These proteins may also be direct targets of TRIM59. As there was no more information about the other proteins, whether they are the direct targets of TRIM59 needs further exploration.

In the present, we revealed that TRIM59 was highly expressed during PH, which may due to the transcription modulation of YAP1/TEAD4. In addition, we also illustrated that TRIM59 may contribute to the pathogenesis of PH via modulating protein ubiquitylation, indicating that TRIM59 may have the potential to be a promising target for the treatment of PH. This study revealed the role of TRIM59 in PH. It complements our understanding about the molecular mechanisms of PH, which will be beneficial to PH treatment.

## Data Availability

The datasets used and/or analyzed during the current study are available from the corresponding author upon reasonable request.
